# Quantifying Mesoscale Neuroanatomy Using X-Ray Microtomography

**DOI:** 10.1523/ENEURO.0195-17.2017

**Published:** 2017-10-16

**Authors:** Eva L. Dyer, William Gray Roncal, Judy A. Prasad, Hugo L. Fernandes, Doga Gürsoy, Vincent De Andrade, Kamel Fezzaa, Xianghui Xiao, Joshua T. Vogelstein, Chris Jacobsen, Konrad P. Körding, Narayanan Kasthuri

**Affiliations:** 1 Department of Biomedical Engineering, Georgia Institute of Technology and Emory University, Atlanta, GA, 30332; 2 The Johns Hopkins University Applied Physics Laboratory, Laurel, MD, 20723; 3 Dept. of Computer Science, The Johns Hopkins University, Baltimore, MD, 21218; 4 Dept. of Neurobiology, University of Chicago, Chicago, IL, 60637; 5 Dept. of Physical Medicine and Rehabilitation, Northwestern University, Chicago, IL, 60611; 6 Sensory Motor Performance Program, Rehabilitation Institute of Chicago, Chicago, IL, 60611; 7 Advanced Photon Source, Argonne National Laboratory, Lemont, IL, 60439; 8 Department of Biomedical Engineering, The Johns Hopkins University, Baltimore, MD, 21205; 9 Institute of Computational Medicine, The Johns Hopkins University, Baltimore, MD, 21218; 10 Department of Physics and Astronomy, Northwestern University, Chicago, IL, 60208; 11 Department of Biomedical Engineering, University of Pennsylvania, Philadelphia, PA, 19104; 12 Center for Nanoscale Materials, Argonne National Laboratory, Lemont, IL, 60439

**Keywords:** Automated segmentation, cell counting, electron microscopy, neocortex, neuroanatomy, X-ray microtomography

## Abstract

Methods for resolving the three-dimensional (3D) microstructure of the brain typically start by thinly slicing and staining the brain, followed by imaging numerous individual sections with visible light photons or electrons. In contrast, X-rays can be used to image thick samples, providing a rapid approach for producing large 3D brain maps without sectioning. Here we demonstrate the use of synchrotron X-ray microtomography (µCT) for producing mesoscale (∼1 µm ^3^ resolution) brain maps from millimeter-scale volumes of mouse brain. We introduce a pipeline for µCT-based brain mapping that develops and integrates methods for sample preparation, imaging, and automated segmentation of cells, blood vessels, and myelinated axons, in addition to statistical analyses of these brain structures. Our results demonstrate that X-ray tomography achieves rapid quantification of large brain volumes, complementing other brain mapping and connectomics efforts.

## Visual Abstract

**Figure:**
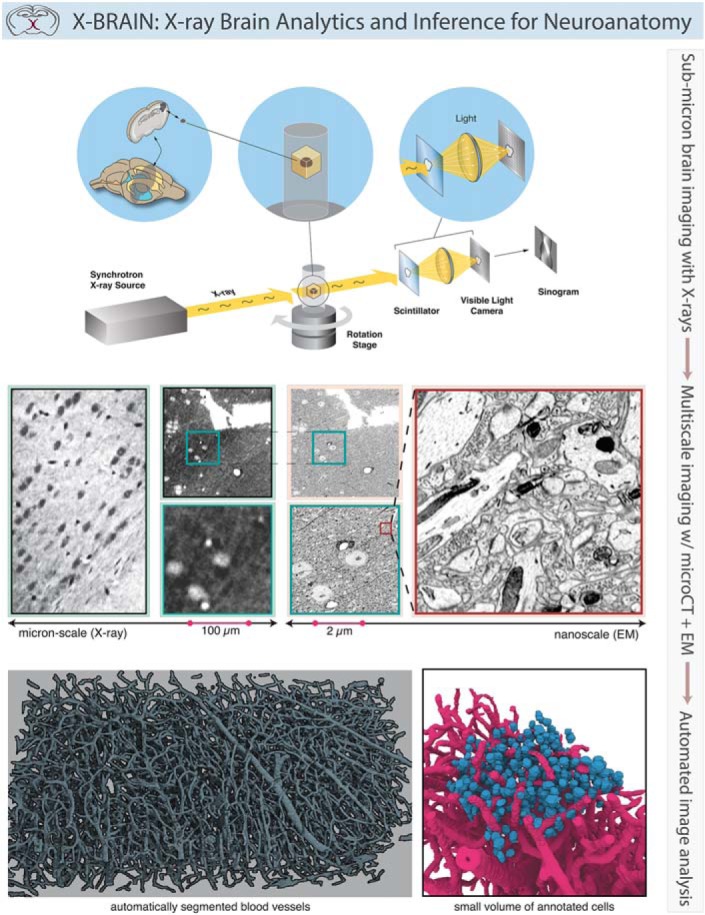

## Significance Statement

Reconstructing neuroanatomical samples in three dimensions is challenging, as traditional methods require fine sectioning of tissue and alignment of these sections into a 3D volume. In this article, we present a pipeline for quantifying neuroanatomy with synchrotron X-ray microtomography (µCT), a method that achieves micron resolution over thick millimeter-scale intact samples. As brain tissue can be imaged with µCT without damaging the integrity of the sample, electron microscopy was applied to survey higher-resolution structures. We introduce this data analysis pipeline for blood vessel segmentation and cell detection, as well as producing estimates of cell densities and spatial relationships among cells and blood vessels. These methods promise efficient imaging, reconstruction, and analysis of brain structures using µCT.

## Introduction

Visualization and quantification of the three-dimensional (3D) microstructure of the brain is integral for constraining models of neural computation, and further understanding how anatomy changes as a consequence of aging and disease (Tsai et al., 2009; Lichtman and Denk, 2011). Traditional brain mapping methods have relied on measurements from small numbers of neurons within a limited number of regions (Yanagihara et al., 1987; Jasmin et al., 1997; Timbie and Barbas, 2015). Recent technologies, in contrast, are rapidly scaling up in their ability to map large neural volumes and eventually interrogate entire brains (Li et al., 2010; Gong et al., 2013; Economo et al., 2016). In all of these efforts, methods for providing high-throughput and unbiased quantification of the brain’s structure are critical.

Although conventional neuroanatomical methods remain essential in neuroscience, these approaches are methodologically overwhelming. For example, light and electron microscopy (EM) methods require large volumes of tissue to be sectioned with micrometer-scale precision, after which these delicate samples must be imaged, stitched, and aligned to reconstruct a full brain volume (Kasthuri et al., 2015; Richardson and Lichtman, 2015). The slicing and stitching requirements of these methods inevitably lead to mechanical distortion within the thousands of samples collected, thereby complicating the reconstruction of the volume. It is only once these images are integrated that cellular and vascular architecture of the brain can be reconstructed at the 3D level. Acquiring high-resolution brain maps using these traditional anatomic methods therefore remains a meticulously demanding and time-intensive endeavor.

More recently, methods such as serial two-photon tomography (Oh et al., 2014), CLARITY (Chung and Deisseroth, 2013; Richardson and Lichtman, 2015), and expansion microscopy (Chen et al., 2015) have been used to visualize details across large neural volumes and in some cases, whole brains. These and other anatomic methods typically introduce external agents (i.e., histologic stains, injected tracers, hydrogels) to define cytoarchitecture or map projections of specific populations of cells (Kreutzberg, 1984; Madisen et al., 2010). Although such approaches have proven incredibly powerful in revealing subsets of brain circuitry with high degrees of specificity (Chung and Deisseroth, 2013; Economo et al., 2016), approaches that provide exhaustive and unbiased visualization of complete neural structures (i.e., all neurons and vasculature within neocortex) are needed to complement these approaches (Mitra, 2014).

EM is presently the gold standard for unambiguous and dense reconstructions (Lichtman and Denk, 2011) of neuroanatomy. But even the newest generation of EM automation (Briggman and Bock, 2012), combined with high-speed EM imaging (Eberle et al., 2015), is limited to investigations of smaller (<mm^3^) volumes of brain tissues by the enormous size of data required to reconstruct even small volumes (Mikula and Denk, 2015). Currently, imaging a cubic millimeter of brain tissue at 20-nm-voxel resolution takes ∼3 months (Eberle et al., 2015) of continuous imaging with multimillion-dollar multibeam scanning electron microscopes, with the resulting data requiring ∼2 petabytes of storage. At these rates, imaging and reconstructing a single cubic millimeter of brain tissue (much less multiple samples from many brains) with EM remains a significant hurdle. Approaches that allow primary mesoscale mapping with subsequent serial EM of the same samples could provide the synaptic resolution of EM in the context of mesoscale maps of large volumes or entire brains.

X-ray microtomography (µCT) provides a largely untapped opportunity for producing dense mesoscale brain maps, in a manner compatible with higher-resolution methods such as EM and nanoCT (De Andrade et al., 2016). Specifically, X-rays penetrate millimeter-scale brain volumes with isotropic micron resolution, thereby removing the need for sectioning tissue (Mizutani et al., 2010a, 2010b; Mizutani and Suzuki, 2012; Zhang et al., 2015; Hieber et al., 2016). Sample integrity and quality of consequent reconstructions therefore remain uncompromised relative to other large-scale visualization methods (Bushong et al., 2015).

Synchrotron-based µCT offers significant photon flux and thus provides efficient acquisition of large brain volumes, two orders of magnitude faster than benchtop µCT systems for neuroscience (Arillo et al., 2015). These advantages have resulted in a quickly growing field of synchrotron X-ray in neuroscience. Synchrotron sources have now been used successfully in the visualization of cerebrovasculature of a whole brain at 6-µm resolution (Zhang et al., 2015), to count cells in cerebellum (Hieber et al., 2016), and to visualize neural networks in spinal cord (Fratini et al., 2015). At present, however, synchrotron-based µCT has yet to be adapted to meet the demands of large-scale brain-mapping efforts. Presently, there are no accessible pipelines which provide methods for transforming X-ray projection images to segmented volumes for the quantification for mesoscale anatomy.

In this article, we introduce a pipeline for rapidly quantifying dense mesoscale neuroanatomy using synchrotron µCT. We demonstrate that samples fixed with aldehydes, stained with heavy metals, and embedded in plastic can be imaged with high-energy synchrotron radiation. The resulting anatomic datasets provide both exceptional isotropic resolution (∼1 µm^3^) and contrast, permitting identification of the 3D architecture of neurons and glial cell bodies, vasculature, segments of large apical dendrites, and myelinated axons. After X-ray imaging, a segment of the large millimeter-scale sample is sectioned and imaged using automated EM, with resulting images displaying excellent preservation of tissue ultrastructure and straightforward correspondence (efficient coregistration) between X-ray and EM datasets. To quantify mesoscale neuroanatomy, we developed an open-source pipeline for X-ray data analysis known as X-BRAIN (X-ray Brain Reconstruction, Analytics and Inference for Neuroanatomy; nerdslab.github.io/xbrain). This set of tools permits efficient blood vessel and axonal segmentation, cell detection, and statistical analyses of X-ray image volumes. µCT in combination with image parsing techniques offers an effective path from brain specimens to mesoscale brain maps.

## Materials and Methods

### Sample preparation

All animal procedures described were performed in accordance with institutional animal care committee regulations. The neocortical sample was prepared using techniques originally developed for large-volume EM. Specifically, an adult female BALB/c mouse was anesthetized with sodium pentobarbital (40 mg/kg) before being transcardially perfused. The vasculature was flushed using 0.1 m cacodylate buffer followed by fixatives (2% paraformaldehyde and 4% glutaraldehyde). The brain was then dissected from the skull, postfixed overnight at 4°C, and sliced on a vibratome at a thickness of 500 µm. After dissection of the somatosensory cortical sample, the tissue was stained with heavy metals in anticipation of subsequent electron microscopy (“ROTO”; Tapia et al., 2012), dehydrated, and embedded in plastic Epon. Anatomical landmarks were used to verify the excised sample was within the range of somatosensory cortex.

### Confirmation of cellular structures with EM

After sample preparation, we used synchrotron-based µCT to image 3D volumes of brain tissue at micron isotropic resolution. We subsequently made ultrathin sections of this tissue using an established approach for automated EM (Kasthuri et al., 2015) to collect low-resolution EM micrographs (∼100-nm pixel resolution). In these low-resolution images, we identified equivalent cell bodies and vasculature localized in the corresponding volume of X-ray data. Fine-resolution EM micrographs (3-nm pixel resolution) were then collected to identify synapses in the EM volumes. Because these labeling approaches are species independent (i.e., they do not require using transgenic animals), we can apply this approach to human and other brain biopsies.

### X-ray data collection and reconstruction

To collect the µCT dataset described here, we used the 2-BM beamline at the Advanced Photon Source (APS). Interested parties who wish to acquire their own X-ray data from the APS through either 2-BM or the 32-ID beamline can submit a General or Partner User Proposal (https://www1.aps.anl.gov/Users-Information/About-Proposals/Proposal-Types for further information). The dataset in this paper was collected with exposure times of 0.1 s per projection and 3000 projections at 30 keV. The 2-BM beamline was equipped with a 10-µm-thick LuAG:Ce scintillator to convert propagation-enhanced X-ray wave into visible light. A microscope objective magnified this signal onto a visible light-scientific CMOS camera (pco.edge 5.5 camera, 2560 × 2560 pixels). When using a 10× objective, this yielded a projection with a pixel size of 0.65 µm. We used propagation-based phase contrast X-ray imaging to obtain high-contrast tomograms of a millimeter-sized region of plastic-embedded and metal-stained mouse neocortex. Imaging a 1-mm ^3^ volume at 0.65 µm isotropic took ∼6 min and required no postacquisition volume alignment or registration processing. The X-ray energy bandwidth was ∼ 300 eV, which means that the data are largely free of the “beam hardening” effects that otherwise complicate medical imaging using laboratory X-ray sources. We are thus able to obtain data ∼130 times faster than with laboratory sources, and with potentially higher image quality.

### Reconstruction of 3D volumes

Datasets were collected in hierarchical data format (HDF) using the Data Exchange schema developed for synchrotron data (De Carlo et al., 2014). Data processing and image reconstructions were performed using the TomoPy toolbox, an open-source Python package, developed at the APS for tomographic data analysis (Gürsoy et al., 2014). We first normalized the projection images with the incident X-ray measurements to suppress artifacts originating from imperfections in the detection process. A wavelet-Fourier filter (Münch et al., 2009) was used to further suppress these artifacts with 10 wavelet levels and an offset suppression value of 2. We used a Paganin-type single-step phase retrieval algorithm to retrieve the phase of the transmitted X-ray signal (Paganin et al., 2002). The location of the rotation center was estimated either automatically, using an optimization approach minimizing the entropy in reconstructions (Donath et al., 2006), or manually, if signal-to-noise (SNR) levels were high. The tomographic reconstructions were performed using the GridRec algorithm (Dowd et al., 1999), which is a fast implementation of the conventional filtered-back-projection method (Kak and Slaney, 1988).

### Preprocessing of image stacks

Each image reconstructed in TomoPy is 2560 × 2560 pixels (0.65 µm isotropic) and is initially stored with 32-bit float precision. We used the multiple image processor tool in Fiji (ImageJ; Schindelin et al., 2012) to color-correct the images by applying automatic contrast enhancement to the image volume and converting the bit depth of each µCT image to 8 bits. By computing the average number of bits of information in each pixel of the original image, we confirmed that an 8-bit depth was sufficient to capture the information in the µCT stack. Visual inspection also confirmed this choice of bit depth, with no visible loss of data quality due to quantization. We then applied an automatic contrast enhancement filter to each image in the stack in Fiji. After reducing the bit depth and masking the data, the dataset was reduced by a factor of 10 (95 GB to 10 GB).

### Volume of the analyzed sample

The image volume that we analyzed in this paper is of size 1400 × 2480 × 1547 voxels, which corresponds to a volume of size 910 × 1612 × 1005 µm (1.474 mm^3^). At this scale, we can adequately test our methodology and find large-scale results that correspond well with previous studies (Tsai et al., 2009).

### Evaluation metrics

To compute interrater reliability and evaluate the performance of our automated methods, we developed tools to compare segmentations at both pixel and object level. Detected pixels/objects that do not appear in the manual segmentation are counted as false positives, and manually identified pixels/objects not found by the automatic segmentation algorithm result in false negatives (misses). In all of our evaluations, we compute precision (*p*), recall (*r*), and f*_β_* score as

$$f_{\beta }=\left(1+\beta^{2}\right)\frac{pr}{\beta^{2}p+r},$$

where we set β = {1,2}. When evaluating the performance of our methods for detecting cells (object-level errors), we compute matches between two sets of centroids by identifying cell pairs in different segmentations that are nearest neighbors. If the matching centroids are within a fixed distance (10 µm) from one another, we label them a match and remove both cells from the dataset to avoid duplicate assignments. The matching process iterates until all possible matches are found, and precision and recall metrics are computed. For cell detection, we computed the *f*_1_ score, as it places equal weight on precision and recall. However, in the case of the pixel-level segmentation of vessels, we observed that optimizing the *f*_2_ score produced more accurate results (as confirmed by visual inspection performed by a trained annotator; see details below).

### Manual annotations and ground truthing

To obtain a ground truth dataset to quantify the performance of our algorithms and assess interrater reliability, we instructed a total of four trained annotators (A0, A1, A2, A3) and five novices to label different subvolumes (V0, V1, V2, V3) of our image dataset using ITK-Snap (Yushkevich et al., 2006). To denote each annotation, we list the annotator and volume; for instance, V2-A0 refers to A0’s annotation of V2. When two annotator IDs are used, e.g., V2-A12, this implies that both annotators (A1 and A2) iteratively refined a common annotation. Most of the subvolumes were selected to produce significant variability compared with previously selected subvolumes, except for V3. Because V3 serves as a final test set, we had an external party blind to the sample’s properties randomly select a subvolume to be used as a held-out test volume (V3) at a location unknown to the authors.

### Interrater reliability

Two of the trained experts (A0 and A1) and the five novices labeled cells and vessels in V1, a 195 × 195 × 65-µm cube of data (300 × 300 × 100 voxels). Annotator A0 was instructed to produce a saturated reconstruction of V1, where all cells and vessels (and their boundaries) were fully labeled. A1 produced a saturated segmentation of a subvolume of V1, denoted V0. To estimate interrater reliability across annotators, we computed the voxel-wise precision and recall between V0-A0 and V0-A1, which we computed to be (*p*,*r*) = (0.93,0.58) for cell bodies and (*p*,*r*) = (0.99,0.29) for vessels. Although precision is high in both cases, the recall is much lower. This is because A1 produces an underestimate of A0’s labels; we tested this by dilating A1’s labels until we maximized the *f*_1_ score between the two annotations (A0 is considered ground truth). In this case, we obtain a precision (*p*,*r*) = (0.84,0.76) for cell bodies and (*p*,*r*) = (0.85,0.73) for vessels.

We then computed the agreement between these annotators in detecting cell centroids. We first processed each segmentation to ensure that each cell is represented by a distinct cluster of pixels, then applied a connected component algorithm to estimate the centroid of each cell. Centroids were then matched across the two annotations to compute object-level precision and recall. When ignoring detections along the boundaries of the volume, no cells were identified by A1 that were not identified by A0, and only one cell was identified by A0 that was not identified by A1. Thus, interrater reliability is nearly perfect when annotators were asked to identify cell centers.

To quantify the time required to label the centroids of cell bodies, we recruited five subjects with no prior anatomic labeling experience to identify the centers of cell bodies in three dimensions. Each subject was instructed to label as many cells as possible in 30 min. The average number of cells that these subjects labeled was 51.2 (median was 62). These results suggest that a novice can accurately (as confirmed by A0) label the centroids of ∼100 cells in 1 h. In practice, we find that it takes all expert annotators (A0, A3) ∼5 h to reliably label all cell centers in a 100-µm^3^ volume. From estimates of the cell density in mouse neocortex, we expect ∼120,000 cells per cubic millimeter. Therefore, manual annotation of every cell in a similar 1-mm^3^ sample would require an estimated 1200 person-hours, or an single anatomist working 24 h/d over the course of 50 days.

### Computing the effective image resolution

To compute the effective resolution of our imaging system, we computed the signal and noise power spectra (SPS and NPS) by taking a series of 256 *xy* transverse planes (i.e., normal to X-ray source) and vertical planes (virtual slices) and averaging their power spectra to measure the resolution parallel to and perpendicular to the rotation axis. We then fitted a second-order polynomial to the SPS to account for artifacts introduced during phase retrieval. When measuring the gap between the smoothed power spectra and the NPS, the signal is five times higher than the noise (following the Rose criterion for detectability; Rose, 1946) at a spatial frequency of 0.383 µm^–1^ in *xy* and 0.525 µm^–1^ in *xz*. This indicates a half-period spatial resolution of 1.31 µm in *xy* and 0.95 µm in *xz*.

### Computing SNR

To estimate the intrinsic difficulty of separating cells from their background, we calculated the ratio of the intensity between cells and their exteriors. To do this, we sampled 10 cells every 25 slices (15.6 µm) in each of the three manually annotated volumes (V1, V2, V3) using ITK Snap. This sampling strategy was chosen to ensure that we had sufficient separation between measured cells. Our protocol required the placement of a small circular marker within the cell’s membrane and another marker external to the membrane (where the cell’s boundary is clearly resolved). This generated 30 samples in both V1 and V2 and 89 samples in V3, providing a measurement of intracellular brightness (signal) and external background (noise). We then computed the SNR for the *i*th cell as *SNR* = 20 log_10_(*s_i_*/*n_i_*), where *s_i_* (signal) and *n_i_* (noise) are the mean value of the labeled pixels within and outside of the *i*th labeled cell, respectively. The mean and SD of the SNR (dB) across each subvolume is: V1 = (4.73, 0.69), V2 = (4.59, 1.49), V3 = (4.49, 1.13). We observed the largest variance in SNR in V2 and the lowest average SNR in V3, with training volume V1 possessing the highest mean and lowest variance SNR in comparison. Our estimates of the SNR are predictive of the difficulty of the segmentation task, and therefore correlated with the accuracy of our segmentation results across a range of neocortical volumes.

### X-BRAIN: Methods for segmenting and analyzing X-ray image volumes

We now provide an overview of the modules and tools provided in X-BRAIN.

#### Step 1: Computing probability maps with ilastik

The first step of our segmentation pipeline is to perform pixel-level classification on the X-ray images (in 3D) to estimate the probability that a voxel is a cell, vessel or lies in the background (other). We used a tool called *ilastik*, which trains a random forest (RF) classifier using sparse (manual) annotations of class labels in the data. *Ilastik* provides an interactive method to compute and examine feature channels; using this interactive mode, we selected a variety of patch-based edge and texture features at different scales to train a pixel-level classifier. In general, we found that intensity features were too sensitive to fluctuations in brightness throughout the sample, and the most useful features were typically the gradient of Gaussian magnitude, difference of Gaussians (DoG), and the structure tensor eigenvalues. To produce probability maps, we developed a python interface to run trained *ilastik* classifiers on volumes of X-ray images.

#### Step 2: Vessel segmentation

After computing the vessel probability map with *ilastik*, we threshold the probability map, dilate the resulting binary thresholded output, and remove spurious connected components based on a minimum size threshold. After applying these simple morphologic filtering operations, we found that the resulting segmentation has better agreement with the manually segmented ground truth than labels produced by a second manual annotator. Thus it appears that vessel segmentation is a relatively easy task once the data has been preprocessed with *ilastik*.

#### Step 3: Cell detection method

Although a RF classifier provides a good starting point for localizing cell bodies, overlapping neurons and vessels are often hard to distinguish by simply thresholding the probability map or using other off-the-shelf cell detection methods such as those provided in *ilastik*. To separate these components, we exploit the fact that most cells in the cortical sample can be well approximated with a spherical shape. Thus we developed a greedy approach, which is similar in spirit to matching pursuit algorithms for sparse signal recovery (Davis et al., 1997), to iteratively refine our estimate of new cell positions and then “remove” newly detected cells from the probability map. At each step of the algorithm, we apply a 3D fast Fourier transform to convolve the cell probability map with a spherical template of diameter roughly equal to that of an average cell (see Step 4 for more details on how we selected the template diameter). After convolving the probability map with the template, we select the global maxima as the centroid of the next detected cell. After finding this cell, we then zero out the probability map in this region to prevent false positives (such that a candidate cell in the same location will not be repeatedly selected). This matching procedure is then repeated until convergence, defined as the point at which the correlation between the probability map and our template drops below a user-specified threshold or reaches the maximum number of iterations.

#### Step 4: Hyperparameter searches

We developed a tool to run hyperparameter searches to maximize the performance of our methods on a densely annotated ground truth volume (V1). After exploring the parameter space, we ran a grid search over the most critical parameters (cell size, dilation, and threshold cutoff) to find a stable, optimal point. We selected the parameters (cell size 18, dilation 8, threshold 0.47) that maximized the *f*_1_ score. Because voxels on the edge of volumes are inherently ambiguous in detection for both human and machine annotators, we choose to disregard these objects in both detected and truth volumes when computing precision and recall scores.

#### Step 5: Nonparametric density estimation

To compute the density of detected cells within a volume, we applied a *k*-nearest neighbor (kNN) density estimation algorithm (Loftsgaarden et al., 1965; Póczos and Schneider, 2011), which estimates the density using only distances between the samples (cells) and their *k*th nearest neighbor. More concretely, we define the distance between a centroid vector $x\in {\mathbb{R}}^{3}$ and a matrix ***A*** as

$$\rho_{k}\left(x,A\right)={\left\|x-a_{k}\right\|}_{2}^{2},$$

where **a***_k_* is the *k*th nearest neighbor to x contained in the columns of A. The value of the empirical distribution *p* at v=(x,y,z) is then estimated using the following consistent estimator (Póczos and Schneider, 2011):

$$p(v)\propto \frac{k}{N\rho_{k}\left(v,V\right)},$$

and V contains the centroids of the rest of the detected cells in the sample. We compute this quantity over a 3D grid, where the volume of each bin in the sample grid is Vol = 8.44 µm^3^. We selected this bin size to ensure that detected cells will lie in roughly a single grid point. This choice was further confirmed by visually inspecting the resulting density estimates. After computing the density for each 3D bin in our selected grid, we normalized these density estimates to obtain a proper probability mass function. Finally, we computed an estimate of the number of cells per cubic mm as $p_{d}\left(v\right)=\left[p\left(v\right)N/\text{Vol}\right]\times 10^{9}$. The rationale behind this approach is that in regions with a higher density of samples, the quantity $\rho_{k}\left(v_{i},V\right)$ will be very small, and thus the probability of generating a sample at this location is large.

### Details of experiments on large-scale datasets

After validating and benchmarking our algorithms, we scaled our processing to the entire dataset of interest (*x* voxels, 610–2010; *y*, 1–2480; *z*, 390–2014; resolution, 0), using the processing environment (Rex et al., 2003). Leveraging, a distributed pipelining environment, allowed us to quickly build interfaces to algorithms written by different research groups or in different languages to assemble a cohesive implementation. These algorithms have well-defined interfaces and can be repackaged for use in a different meta-scheduler environment. When running at scale, we first divided our large data volume into small cuboids meeting our computational constraints. A spatial database was used to get and store data; image data were requested for each computed block, and the results were written to a spatially coregistered annotation channel (Burns et al., 2013; Kleissas et al., 2016). Each block was retrieved with sufficient padding to provide edge context; we processed these blocks in a parallel fashion and uploaded the resulting detections. We also implemented an alternative merging strategy to account for cells near boundaries, by eliminating putative detections that touch a block edge or overlap with existing objects in the spatial database. This was implemented to further reduce edge effects and thereby minimize the possibility of false positives.

### Axonal reconstruction

To segment small branches of myelinated axons (Fig. 8*a*), we applied the same preprocessing steps as before and trained a new *ilastik* classifier to segment blood vessels, cells, and axons from background. After retraining the classifier to segment axons, we applied the *ilastik* classifier to the same small 333 × 333 × 130-µm volume and applied the same techniques previously used for vessel segmentation to segment the axons in the sample. We thresholded (*p* < 0.3), eroded, and dilated the axonal probabilities using a spherical structuring element of size 4, and then applied a connected component algorithm to label each connected component with a different ID.

### Data accessibility and reproducibility

All of our algorithms, data, and data derivatives are open source and available for those in the neuroscience community to reproduce and leverage for further scientific discovery. Both our raw data and its derivatives are freely available for download and visualization (nerdslab.github.io/xbrain). To facilitate reproducibility, scripts to download data and annotations and generate the figures from this article are provided in both Matlab and Python. To create the 3D visualizations described, we relied on a script that interacts with the spatial database and pulls down an .obj file suitable for processing with the open-source Blender tool.

## Results

### X-ray tomography of a millimeter-scale brain sample

**Fig. 1.**
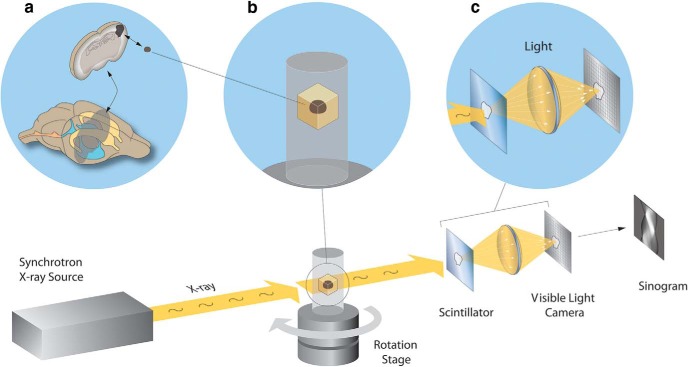
Synchrotron X-ray tomography of a millimeter-scale brain sample. A schematic illustration of the imaging setup is displayed along the bottom: from left to right, the synchrotron X-ray source interacting with an embedded sample of somatosensory cortex as it is rotated during the collection of multi-angle projections. To collect this projection data, X-rays are passed through a scintillator crystal that converts the energy into visible light photons. These photons are then focused onto a light camera sensor, before a sinogram is generated via data collection from a row of sensor pixels. In the three panels above, visualizations of the neocortical sample preparation (***a***), location of the mounted sample within the instrument (***b***), and conversion and focusing of X-rays into light photons (***c***).

Using the 2-BM synchrotron beamline at APS (De Carlo et al., 2014), we obtained tomography data from a volume of neocortex (Fig. 1) prepared using methods compatible with subsequent large-volume EM (Tapia et al., 2012; see Methods). Stacks of projection images were acquired by rotating the sample (Fig. 1*a*) at 3000 uniformly spaced angles from 0 to 180 degrees and measuring the propagation of X-rays through the sample at each rotation angle (Fig. 1*b*). Radiographs are recorded with an indirect detection system consisting of a thin scintillator that converts the transmitted X-rays into visible light (Fig. 1*c*). The light is then focused by an objective lens on a charged coupled detector (CCD) array, producing images with equivalent pixel size of 0.65 µm^2^ at the sample plane. Collecting the dataset studied in this paper (10.6 Gigavoxels) took ∼6 min. To obtain high-contrast images, data acquisition was performed in propagation-based phase contrast mode by increasing the distance between the detector and sample to several tens of centimeters and imaging with a pink beam (ΔE/E = 10^–2^) set to 30 keV. To reconstruct a 3D image volume from the projection data, phase retrieval was performed on each projection using the well-established Paganin algorithm (Paganin et al., 2002; Weitkamp et al., 2011), followed by volume reconstruction using the open source TomoPy package (Gürsoy et al., 2014). The resulting image volume provides the data for our segmentation and analysis methods.

**Fig. 2.**
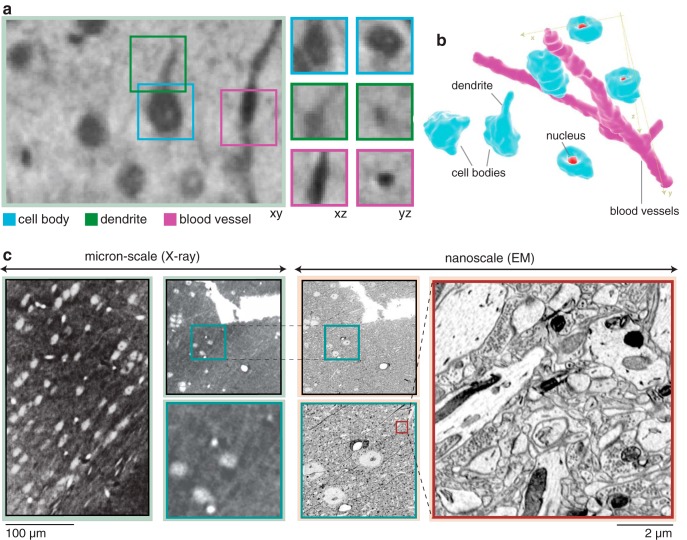
Synchrotron X-ray imaging provides micron resolution within a neocortical volume. ***a***, Microscopic visualization of cells, blood vessels, and dendrites within an X-ray–imaged volume of somatosensory cortex. Each panel shows one of three perspectives within the *xyz* coordinate framework (panels to the right are 11.5 µm wide, large panel to the left is 100 µm wide). ***b***, Digital rendering of a manually reconstructed subset of blood vessels and cell bodies (nuclei highlighted) selected from within the neocortical volume. ***c***, Photomicrographs of a subvolume within this sample, using µCT and EM to identify overlapping regions. These images were collected at three different pixel sizes (0.65 µm, 100 nm, 3 nm). In the left panel, a subset of a single virtual slice from an X-ray tomogram that spans the neocortical volume (0.65 µm pixel size). Outlined in blue to the right of this is a subset of the volume (within ***a***) that highlights a configuration of three cell bodies and distinct proximal microvessels. This sample was subsequently serially sectioned and imaged in a scanning electron microscope. These cells are located in the EM dataset (inset), the ultrastructure of which is well preserved, even after µCT (right in red).

To quantify the resolution of our reconstructed X-ray image volumes, we obtained digitally vignetted subfields from regions with brain tissue (signal) and without (background) and computed their respective 2D Fourier power spectra (see Methods for further details). This power spectral analysis reveals nearly isotropic resolution and an effective resolution of 1.2 µm. At this resolution, X-ray images allow resolving the putative location and size of cell bodies and their nuclei, blood vessels, and segments of large neurites (Fig. 2*a*,*b*). We estimate that voxels inside cells are on average 4.56 ± 1.13 dB (mean ± SD) brighter than voxels in the immediate surrounding region (see Methods). At this contrast level, the location and size of cells in the sample can be unambiguously resolved. Blood vessels are equally visible in this sample and provide even greater contrast than cell bodies, making them highly accessible for quantification. This high signal-to-background strength justified the capacity to segment the sample into cell bodies and blood vessels, which we validate with our automated techniques.

After collecting µCT data, we performed ultrathin sectioning and EM on the same sample. EM confirmed the identity of the cell bodies and their nuclei, blood vessels, and large neuronal processes observed in the µCT dataset (Fig. 2*c*). These data strongly support our observations that the structures identified in the X-ray dataset are anatomically authentic and not spurious consequences of the X-ray imaging pipeline. In addition, there were no deformations in the microtome sectioning properties of the Epon-embedded brain tissue, nor any obvious signs of irradiation-induced structural damage in the scanning electron micrographs obtained from these sections. Structures such as synapses and mitochondria remain clearly evident (Fig. 2*c*). We anticipated the preservation of cellular architecture, given our calculated radiation dose of ∼3 kGy during the collection of the X-ray tomography data. This dose is well below the dose affecting the dissolution rate of radiation-sensitive polymers such as poly(methyl methacrylate) (PMMA; Zhang et al., 1995; 1000 kGy), and the dose at which glutaraldehyde-fixed wet chromosomes start to show mass loss (Williams et al., 1993; 70 MGy). Our results confirm that µCT and EM can be integrated to produce a multiresolution reconstruction of a neocortical volume.

Any high-quality anatomic dataset should permit one to reliably annotate structures of interest. We thus measured human annotator performance in the localization and labeling of cell bodies and blood vessels in multiview projections (orthogonal 2D projection planes) of the 3D image data. Two expert annotators (A0 and A1) were instructed to label the boundaries of every cell and vessel within a small volume (100 µm^3^) of X-ray image data using ITK-Snap, an open-access software tool used to annotate 3D images (Yushkevich et al., 2006). When provided with 3D context, pixel-level agreement (precision, recall) between annotators of the cell bodies and blood vessels was (*p*,*r*) = (0.835,0.76) and (*p*,*r*) = (0.85,0.73), respectively (see Methods). We further measured the consistency of annotator’s ability to localize centroids of cell bodies and found nearly perfect agreement (*p*,*r*) = (1,0.989). Although precise manual segmentation of cell body boundaries and vessels remains challenging, we observed high interrater reliability between annotations of the centers of cell bodies. This demonstrates that human performance on the detection task is nearly identical and suggests that our X-ray–generated dataset is of sufficient quality to be segmented using automated methods.

### Automated methods for segmentation and cell detection in X-ray volumes

**Fig. 3.**
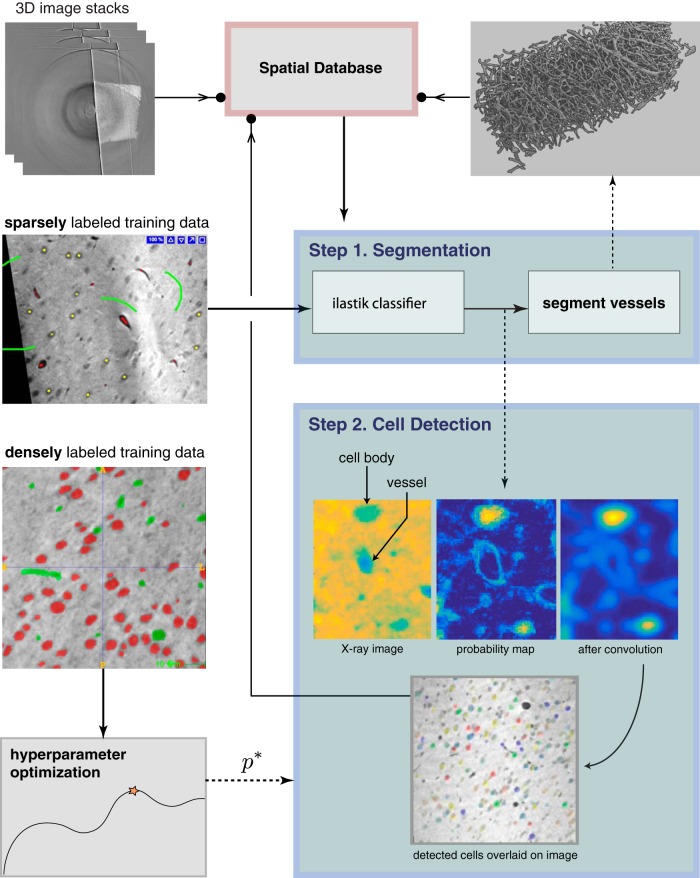
Image processing and computer vision pipeline for segmentation and cell detection. Block diagram displaying the entire X-BRAIN workflow is described. The integration of sparsely labeled training data into our segmentation module (Step 1) is used to train a random forest classifier using *ilastik*. Densely annotated training data are used to perform hyperparameter optimization to tune our cell detection algorithm in Step 2. The final map of detected cells is displayed at the bottom of Step 2, with detected cells overlaid on the original X-ray image. Solid arrows, inputs into a module; dashed arrows, outputs; filled circle terminal, outputs that are stored in the spatial database.

Although annotators A0 and A1 manually reconstructed a subvolume of our sample with a high degree of accuracy, complete datasets afforded by X-ray tomography remain overwhelming in magnitude and density. It is therefore inefficient to densely reconstruct these datasets solely using human input. To analyze large-scale X-ray datasets, we developed automated 3D segmentation algorithms to extract cells and vessels from the resulting X-ray image volumes. We created a suite of tools for extracting and visualizing mesoscale maps from stacks of X-ray images (Fig. 3). This toolkit, which we call X-BRAIN (X-ray Brain Reconstruction, Analytics, and Inference for Neuroanatomy), consists of image processing and computer vision methods for preprocessing and artifact removal, vessel segmentation, and estimation of cell size and location. We also provide methods for large-scale statistical analyses for the resulting reconstructed cell and vessel maps. Matlab and Python code, manual annotations, and image data are openly available through nerdslab.github.io/xbrain, providing a community resource for the automated segmentation and quantification of mesoscale brain anatomy.

After finding the centroids of all detected cells, we can efficiently estimate their sizes. To do this, we center a small spherical template at the detected center of each cell and estimate the cell size by increasing the size of the template. When the template can no longer be inscribed within the cell body, we observe a sharp decay in the correlation. Thus we compute the correlation between the probability map while increasing the diameter of the spherical template, find the maximum decrease in correlation, and select this corresponding diameter as our estimate of the cell size. This operation has low complexity and can be performed on the entire dataset (50,000 cells) on a single workstation. Once cells have been detected, estimating the diameter of the cell body is a simple one-dimensional fitting problem.

**Fig. 4.**
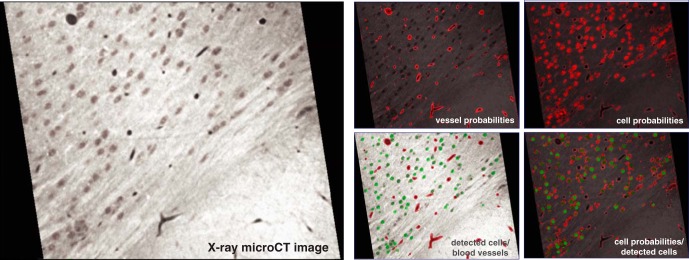
Visualization of X-ray image data, overlaid probability maps, and final segmentations. On the left, an X-ray micrograph. On the right, clockwise from upper left: vessel probabilities, cell probabilities, cell probabilities and segmentations, and the segmentations of cells and vessels.

Our main image processing and computer vision pipeline (Steps 1–2 in Fig. 3) consists of methods for segmenting blood vessels and detecting the location and size of cells in the volume. In the initial step of our workflow, we train a classifier to predict the probability of brain voxels belonging to each of the three classes: cell body, blood vessel, and background (other). To do this, we use the interactive learning and segmentation toolkit *ilastik* to sparsely annotate the dataset and build a random forest classifier using intensity, edge, and gradient features computed on the image volume (Sommer et al., 2011). This classification procedure returns three probability maps $P=\left\{P_{c},P_{v},P_{bg}\right\}$, which collectively provide the probability tuple $p\left(x,y,z\right)=\left\{P_{c}\left(x,y,z\right),P_{v}\left(x,y,z\right),P_{bg}\left(x,y,z\right)\right\}$ that each voxel whose position is denoted by (*x*,*y*,*z*), is a cell, vessel, or lies in the background (output of *ilastik* in Step 1 of Fig. 3, see Fig. 4). This classification procedure provides an accessible and intuitive way to generate an estimate of which voxels correspond to cell bodies and blood vessels.

**Fig. 5.**
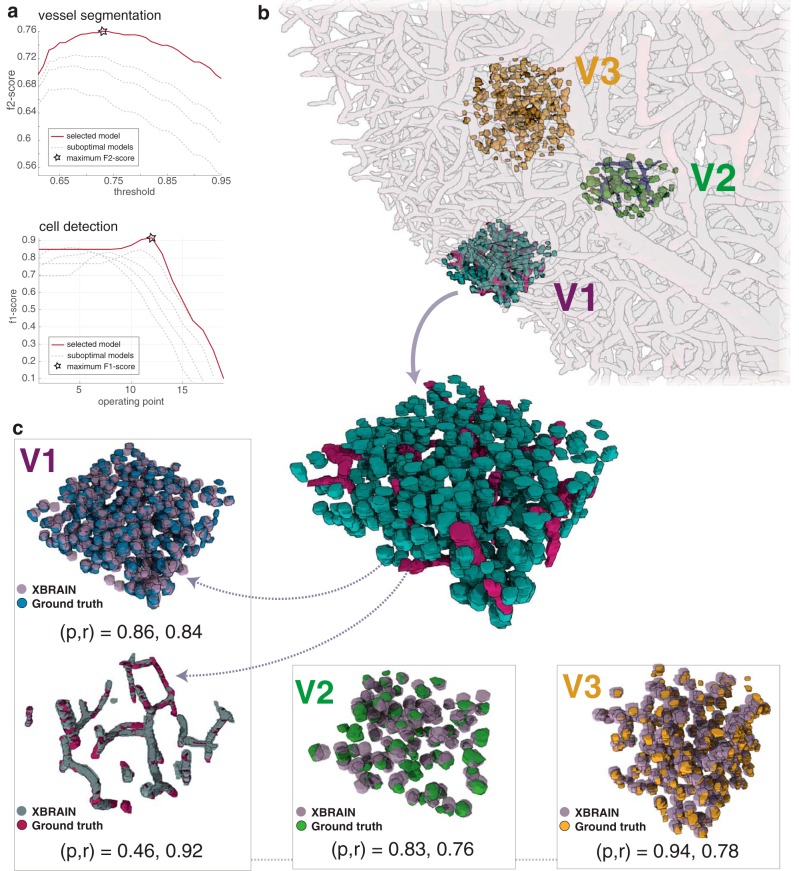
Automated methods for segmentation and cell detection reveal dense mesoscale brain maps. ***a***, Performance of vessel segmentation and cell detection methods, as hyperparameters that affect the performance of the method, are varied. To optimize performance of the vessel segmentation method, the *f*_2_ score is computed—emphasizing recall—for multiple operating points (each curve represents a fixed parameter set with a varying vessel segmentation threshold). To measure performance for cell detection, the *f*_1_ score—balancing precision and recall—is calculated for multiple operating points as the stopping criterion is increased (*x* axis) in the greedy cell finder algorithm. Highlighted curves within each plot and the accompanying “star” indicate optimal hyperparameter performance. ***b***, Results of cell detection and vessel segmentation algorithms on manually annotated test datasets. The training volumes V1 (195 × 195 × 65 µm and V2 (130 × 130 × 65 µm) and test volume V3 (130 × 130 × 130 µm) are visualized within the entire volume of X-ray–imaged tissue. ***c***, Training volumes V1 and V2 and test volume V3 individually visualized. In each manually annotated subvolume, the results of X-BRAIN are overlaid, based on the best operating point selected by the parameter optimization approach in ***a***. The precision (p) and recall (r) values for each subvolume are further annotated.

The simplest way to convert a probability map to a (binary) segmentation is to threshold the probabilities and label each connected component as a discrete object. In the case of vessel segmentation, we successfully use this procedure with minimal tweaks. To segment vessels in the sample, we threshold the vessel probability map and then apply simple morphologic filtering operations to clean and smooth the resulting binary data (see Methods). Visual inspection and subsequent quantification of precision and recall of vessel segmentation (Fig. 5*a*) suggest a high degree of accuracy through this simple postprocessing of the *ilastik* outputs.

Applying the same thresholding procedure used for vessel segmentation to the segmentation of cells is problematic, as neurons and blood vessels are often densely packed in neocortex. The complicated nature of segmenting densely packed data is not trivial (Qi et al., 2012). Therefore, we developed an algorithm for cell detection (Step 2 in Fig. 3) that produces estimates of the centroids and radii of detected cells. Our method iteratively selects a new candidate centroid based on the correlation between the cell probability map and a (fixed-radius) spherical template. We use a frequency-based approach to convolve a spherical template with the cell probability map and greedily select “hot spots” that are likely to contain cell bodies (see Methods). Our method leverages prior biological knowledge of the approximate size and spherical shape of cells to select spherelike objects from the prefiltered probabilities to resolve situations where neurons appear in close proximity to one another.

### Performance evaluation

Understanding the stability and performance of our segmentation method is critical for assessing the accuracy of the maps provided by our approach. Using a densely annotated training dataset (V1), we performed a grid search to find the set of hyperparameters (e.g., threshold parameters for cell/vessel detection, the size of spherical template, and stopping criteria) that maximized a combination of the precision and recall (*f* score) between our algorithm’s output and manually annotated (A0) data from the training volume (Fig. 5*a*). After tuning our cell detection algorithm to find the best set of hyperparameters, we obtained a precision and recall of (*p*,*r*) = (0.86,0.84). The results of our hyperparameter search suggest that our methods are stable and provide good performance across a range of parameters.

To verify that our segmentation algorithm generalizes across regions previously unseen during classifier training, we labeled and tested our cell detection algorithm on two additional test cubes, V2 and V3 (Fig. 5*b*,*c*), that are spatially independent from V1 and each other (see Methods as well as Table 1 for more information about training and test volumes). V2 served an initial test set, as we added some sparse training data from this volume to train our *ilastik* classifier. V3 served as a held-out test set, as the location of this cube was unknown before tuning and running the algorithm on the entire dataset. After obtaining ground truth labels via manual annotation provided by A0/A1, we ran X-BRAIN on both V2 and V3 using the set of parameters selected by optimizing our method on V1. The precision and recall is given by (*p*,*r*) = (0.83,0.76) and (0.94,0.78), for V2 and V3, respectively. These results show that our approach for hyperparameter optimization can be used to obtain accurate cell detection performance when applied to new regions in the sample.

**Table 1.** Statistics of cell counts in manually and automatically labeled volumes

*Annotation*	*Cells,* *n*	*Area*	*Volume (% of mm^3^)*	*Density (10^5^/mm^3^)*
V0-A0	97	(2136, 2060)	0.06	1.63
V0-A1	96	(1489, 1499)	0.06	1.28
V0-Xbrain	94	(1983, 2123)	0.06	1.57
V1-A0	321	(1997, 2035)	2.5	1.28
V1-Xbrain	302	(1983, 1963)	2.5	1.21
V2-A12	103	(1416, 1301)	0.06	1.72
V2-Xbrain	112	(1918, 1963)	0.06	1.87
V3-A03	281	NA	0.2	1.41
V3-Xbrain	240	(1419, 1385)	0.2	1.20
Vtot-Xbrain	48, 689	(1454, 138)	42	1.02

The first column of the table displays the name of the volume (V0, V1, V2, and V3) as well as the annotator: manual annotator (A0, A1, A2, A3) or automated annotation (X-BRAIN). In the second and third columns, the number of detected cells and the area (mean, median) of annotated cell bodies (number of labeled voxels) are described. Volumes (percentage of cubic millimeters) of all the reported subvolumes are in the fourth column. Finally, we report the density of each subvolume in the fifth column. Note that V0, V1, and V2 are all manually annotated volumes used to train and tune our automated methods. V3 is a held-out test set whose location was unknown during training and tuning the parameters of the algorithm. NA, not applicable.

Fluctuations in brightness make the segmentation of X-ray volumes more difficult. To understand the relationship between these fluctuations and the difficulty of the cell detection problem, we computed the SNR at multiple points within each of the labeled volumes. The mean and SD of the SNR (in dB) between cells and their background in all three volumes was V1 = (4.73, 0.69), V2 = (4.59, 1.49), and V3 = (4.49, 1.17). As expected, the precision and recall (for cell detection) appear to be correlated with the variance of the SNR in the volume. We obtain high precision and recall for V1 and V3, and indeed, these volumes exhibit smaller variance in their contrast between cells and their background. Even with of fluctuations in brightness, our results and sensitivity analysis on training and test volumes suggest that X-BRAIN generalizes well across different regions of the volume.

### Large-scale analysis and visualization

**Fig. 6.**
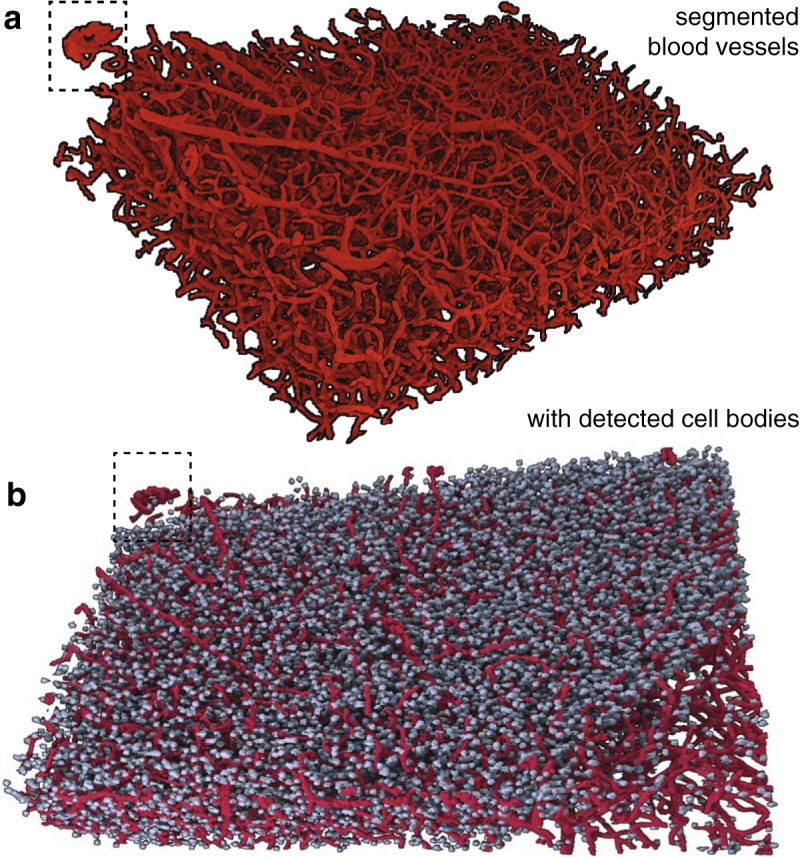
Visualization of 3D reconstructions of the neural architecture within a millimeter-scale neocortical sample. ***a***, Renderings of the vessel segmentation algorithm output across the depth of the entire analyzed sample. ***b***, Visual perspective of the cell detection algorithm output integrated with renderings from vasculature displayed in ***a***, with hatched inset showing the same subset of both neurons and vessels.

**Fig. 7.**
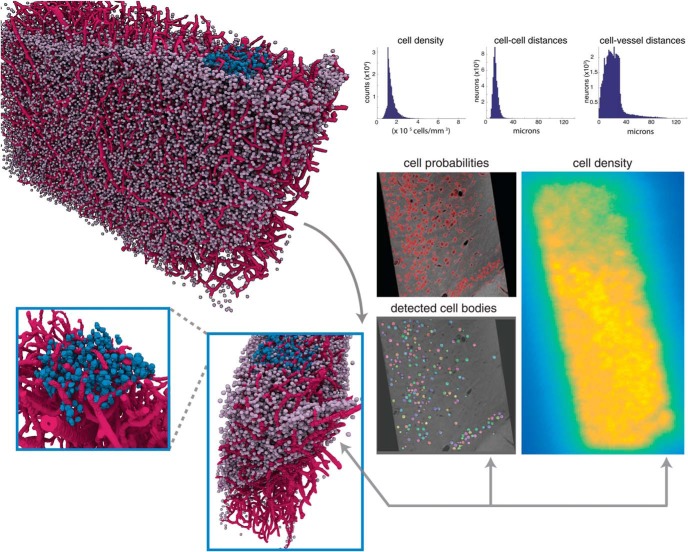
Spatial statistics of X-ray volumes reveal layering and spatially diverse distribution of cell bodies. Top right, histograms of the estimates of the cell density over the extent of the entire sample of mouse cortex, distances between the center of each cell and its nearest neighbor (cell-to-cell distances), and distances between the center of each cell and the closest vessel voxel (cell-to-vessel distances). Top left, 3D rendering of the detected cells and vessels in the entire sample, with a manually labeled cube (V1) highlighted in blue. To confirm the 3D structure of this visualization (bottom left), confirmation is provided in the maps provided to the right: cell probability (red indicating high probability), detected cells (each detected cell displayed in a different color), and density estimates (bright yellow indicating high density). These results provide further confirmation that the 3D structure of the sample is preserved within our density estimate.

To apply X-BRAIN to large datasets, we created an analytics workflow that uses the LONI Pipeline environment (Rex et al., 2003) to automatically distribute jobs across a cluster environment. Our workflow is parallelized by dividing the dataset into small data blocks that can be processed independently, based on a user-specified graphical (xml-based) description of the dependencies between various algorithms. Running our analytics pipeline on a cubic millimeter-scale sample took ∼6 h on a small 48-core cluster (see Methods). As a result, we detected 48,689 cells over the extent of the analyzed sample (2560 × 2560 × 1624 voxels). Furthermore, when we visually inspect our large-scale results (Fig. 6), we find a good correspondence between cells and vessels that are visible to human annotators and those detected by our algorithms. Our initial results on this training volume and visual inspection of the results on the whole sample (Figs. 6 and 7) suggest that our methods provide reliable maps of the cells and vessels in the sample.

One advantage of having isotropic resolution is that we can obtain cell counts and densities in three dimensions. To estimate the 3D density of cells, we applied a robust nonparametric approach for density estimation. Adopting a nonparametric approach enables us to obtain an accurate estimate of the distribution without making any restrictive assumption on its form. In particular, we rely on the popular kNN density estimation algorithm (Loftsgaarden et al., 1965; Póczos and Schneider, 2011), which estimates a distribution using only distances between the samples (cells) and their *k*th nearest neighbor. When applied to the entire volume of our sample, we calculated an average density of 1.3 × 10^5^ cells per mm^3^ (Fig. 7). These results are comparable to other studies that estimate an average of 1.2–2.5 × 10^5^ cells per mm^3^ in mouse neocortex (Tsai et al., 2009), both in terms of our average and the spread of the distribution. Our large-scale analysis provides further evidence that our X-ray pipeline provides accurate estimates of the location and distribution of cells within the sample.

The relative location of cell bodies to each other and vasculature is integral for studying diseases that afflict the brain, particularly in traumatic brain injury and stroke (Werner and Engelhard, 2007; Tsai et al., 2009; Lapi and Colantuoni, 2015). To provide researchers with the tools to quantify these data, we developed automated methods to compute distances between detected cell centers (cell-to-cell distances) and distances between each cell and the closest segmented vessel (cell-to-vessel distances, Fig. 7). Cell-to-vessel distances are spread between 10 and 40 µm, with very few cells exceeding this distance (34.3 ± 533.4 µm). In contrast, cell-to-cell distances appear to be much more concentrated, with a strong peak at 12.7 µm and much smaller variance (21.3 ± 43.1 µm). The distribution of distances between cells and vessels (Fig. 7) aligns with previous results (Tsai et al., 2009; Wu et al., 2014) and confirms the accuracy of our approach. We further estimated that the fractional volume of vessels in the sample as 1.85%, which is in agreement with ranges provided in previous studies (Heinzer et al., 2006; Tsai et al., 2009; Wu et al., 2014). Collectively, our findings reveal the proximity of cells and vasculature within a dense subsampling of the somatosensory cortex.

To complement our suite of analytical tools, we developed methods to produce and visualize mesoscale maps with cellular density and vasculature as their output (Figs. 4 and 7). After running a sample through our pipeline, users can obtain different descriptions of the neuroanatomy combined with the image data to help reveal relevant structures in their output. Using these tools, the user can easily interrogate subvolumes of the data both quantitatively and qualitatively. As an illustrative result, we identified a 3D region of interest comprising deep layers of the somatosensory cortex (see Fig. 7). We confirmed the validity of this structure using multiple avenues: 3D visualizations, X-ray micrographs, cell probability maps, and estimate of cellular density. Each of these representations provide detailed information and descriptions of the data that can be used to further visualize and quantify neuroanatomical characteristics. Our ability to produce maps and generate reconstructions that integrate neuronal cytoarchitecture and vasculature provides neuroscientists with a unique approach to examine neural substrates.

### Axonal reconstruction and tracing

**Fig. 8.**
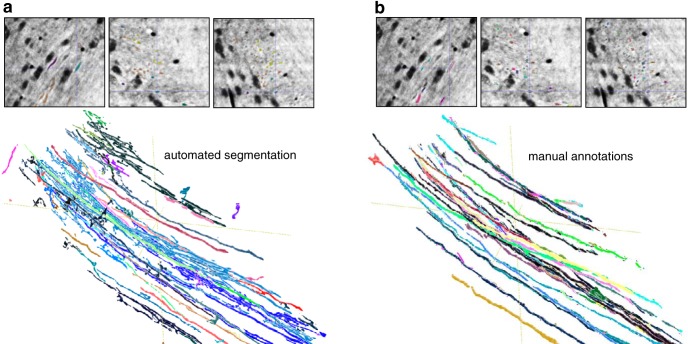
Axonal reconstructions obtained through manual and automated methods yields high agreement. Segmented outputs are overlaid onto X-ray neocortical images (*xy*, *xz*, *yz* planes in the upper panels) and reconstructed in the lower panels for the proposed automated segmentation method (***a***) and manual annotations (***b***).

Mapping long-range, myelinated axons remains an integral feature of traditional neuroanatomical methods. A subset of myelinated axons was found within the corpus callosum at the base of our sample, providing another opportunity to perform automated and manual segmentation. To produce these automated results, we used our pipeline to rapidly retrain an *ilastik* classifier to segment blood vessels, cell bodies, and axons from tissue background (see Movie 1 and Fig. 8*a*). We then applied our approach for vessel segmentation selectively to the dataset’s axons, which were compared to the findings of human annotators (Fig. 8*b*). These results are visually similar, with a greater number of axons detected by the automated approach relative to human annotators (compare lower panels of Fig. 8*a* and *b*). These preliminary findings are unfortunately constrained by the nature of the sample dissection, which has limited the number of axons accessible for reconstruction. When provided with samples with greater axonal density, we anticipate that our pipeline will be able to produce maps of finely traced axons.

**Movie 1.** Myelinated axons, cells, and blood vessels in a small subvolume of neocortex. Each frame of the movie represents a virtual slice through the unsectioned volume, where the pixel size is 0.65 µm isotropic. To create each false-color image in the 3D stack, the raw probability maps for cells and blood vessels are stacked into the image’s green and blue channels, respectively. To visualize myelinated axons of the sample, the probability map is thresholded, and a small fraction of components are removed and then added to the red channel of the image (see Fig. 8*a* for a 3D rendering of these axonal segments).

## Discussion

Here we describe the use of synchrotron-based µCT to efficiently resolve the microstructure (cells, vasculature, and myelinated axons) within a millimeter-scale neocortical sample. Through the integration of traditional and modern anatomic approaches, we have quantified and further validated our µCT generated data. Specifically, we conducted tests of interrater reliability followed by machine-learning methods to segment this data, generating high-quality reconstructions of the sample volume. We have provided our entire suite of open-access resources for the neuroscience community to perform their own µCT neuroimaging data analysis. Finally, we tested our pipeline’s ability to identify myelinated axons and compared this data to manual annotations. Our preliminary findings reveal highly similar reconstructions, which can be expanded on with future densely myelinated datasets. Collectively, our findings showed that automated algorithms can be applied to µCT datasets to rapidly compute3D brain maps with submicron resolution.

The experimental protocol we developed can be used to conduct both µCT and automated EM imaging (Tapia et al., 2012) within the same brain sample. We demonstrate that uniting these techniques provides complementary images of a neocortical volume, without requiring any modification to existing EM preparation protocols. µCT has been previously used with EM in the assessment of sample quality (Bushong et al., 2015; Ng et al., 2016), finding regions of interest to be later investigated at higher resolution (Karreman et al., 2017), and in the alignment and mapping of functional calcium imaging data onto single cells (Bleckert et al., 2016). Whereas these methods focused primarily on the use of X-rays to aid in the analysis of EM data, our work focused on producing high-quality and automated mesoscale maps using X-rays. The dense mesoscale maps afforded by X-BRAIN will further facilitate integration of data generated by X-ray and EM, thereby improving and augmenting approaches for correlating these imaging methods.

The algorithms we developed to parse µCT datasets provide high precision and recall, thereby suggesting that our segmentation and cell detection methods can be used to rapidly and reliably survey data volumes in the localization of cells and vasculature. Thus information about the location and size of cells and vessels, obtained through X-ray image analysis, can be used to improve the alignment of EM datasets and also facilitate merging 2D outputs from segmented EM images (Gray Roncal et al., 2015). Because our open-access pipeline for X-ray image analysis has been integrated using community standard tools and approaches, we can readily combine existing EM analysis pipelines with our methods to analyze a dataset imaged using µCT and EM. These results can be combined to create a multimodal brain map, one providing information about the cytoarchitectural and cerebrovascular properties of a sample, as well as the fine-scale details afforded by EM (e.g., neuronal processes and synapses).

Our segmentation and analysis tools have been developed for analyzing X-ray datasets designed to be compatible with EM. However, we developed this pipeline to be modular and easily applied to datasets from varying imaging conditions. By simply retraining the classifier on a new dataset, our analytics pipeline can be readily applied to analyze other sample preparations for X-ray (Fratini et al., 2015; Hieber et al., 2016), as well as low-resolution EM and other light microscopy approaches. Moreover, because our cell detection method is designed to resolve individual cells that can be tightly packed in cortex (1- to 2-µm separation), we expect that our methods can be beneficial in the analysis of other densely packed brain areas such as the granular layer in cerebellum. We believe the computational methods presented here could provide a scalable approach for analyzing dense reconstructions of the brain’s microstructure across a variety of different imaging conditions and modalities.

Within our neocortical sample, we have resolved nuclei and large cellular processes such as apical dendrites (Fig. 2*a*,*b*). This data can be used to discern whether a cell is indeed a neuron (as opposed to a glial cell). However, to use µCT to reliably identify cell types, the resolution of µCT must be enhanced toward the nanometer scale to permit the resolution of cellular morphology (Peng et al., 2015). To complement this kind of classification, transgenic or immunohistochemical approaches that label distinct neuronal and nonneuronal cell types for µCT must also be developed (Ng et al., 2016). By integrating these approaches in future µCT studies, we can enhance the resolution of reconstructed volumes while providing cellular identification, thereby enabling a more detailed comprehension of brain architecture.

The resolution of µCT is currently limited, as radiation dose increases quadratically with image resolution (Howells et al., 2009). Thus as we increase the resolution, beam damage can induce changes in sample geometry while the tomogram is being acquired, leading to reconstruction artifacts and the degradation of spatial resolution. However, the effect of radiation damage is greatly reduced in cold samples. Thus X-rays have the potential for sub–30-nm resolution 3D imaging of frozen hydrated brain biopsies (Deng et al., 2015) with no chemical modification or plastic embedding. In addition to cooling the sample, additional imaging parameters including photon energy, coherence, and optics can also be optimized to minimize damage to a specimen while increasing image resolution. These imaging parameters can be adjusted to optimize µCT to resolve finer-scale processes and the morphology of neurons.

Limited ground truth data currently restricts the complexity of methods that we can apply to solve our segmentation problem. With more training data from human annotators, we can leverage more sophisticated nonlinear classification strategies such as convolutional neural networks for segmentation and axon tracing. These approaches have been shown to achieve state-of-the-art performance in the identification of synapses and segmentation of cell bodies in EM data (Gray Roncal et al., 2015; Turaga et al., 2010). Finally, improvements in the spatial resolution of tissue samples will aid in the challenge of resolving adjacent neural structures as separate objects, thereby leading to more efficient and robust approaches for cell detection.

Standardized atlases that characterize the macroscale organization of the brain, such as Brodmann maps (Zilles and Amunts, 2010), have been based primarily on neuroanatomists working with thin, sparsely labeled brain samples. However, with developments in large-scale EM connectomics (Helmstaedter et al., 2011; Gray Roncal et al., 2015) and the techniques we present here for µCT, far larger and more comprehensive datasets are possible at a scale previously unachieved. Indeed, it is possible to scale up µCT imaging to large volumes of human brain tissue, such as the cerebellum and frontal cortex (Mizutani et al., 2010b; Hieber et al., 2016). Furthermore, the capabilities of µCT combined with staining approaches for entire brain preparations (Mikula and Denk, 2015) offer the possibility of imaging whole brains at submicron resolution. With these capabilities, generating whole brain maps in a modern data-driven fashion will soon be possible, thereby enabling the massive-scale quantification of the effects of disease, development, and learning in the brain.

## Synthesis

Reviewing Editor: Leonard Maler, University of Ottawa Decisions are customarily a result of the Reviewing Editor and the peer reviewers coming together and discussing their recommendations until a consensus is reached. When revisions are invited, a fact-based synthesis statement explaining their decision and outlining what is needed to prepare a revision will be listed below. The following reviewer(s) agreed to reveal their identity: H. Sebastian Seung.

Thank you. The changes you have made to the manuscript have made it more readable and it may now serve to introduce a wider neuroscience audience to this potentially transformative method.

## References

[B1] Arillo A, Peñalver E, Pérez-De La Fuente R, Delclòs X, Criscione J, Barden PM, Riccio ML, Grimaldi DA (2015) Long-proboscid brachyceran flies in Cretaceous amber (Diptera: Stratiomyomorpha: Zhangsolvidae). Syst Entomol 40:242–267. 10.1111/syen.12106

[B2] Bleckert AA, Brittain D, Borseth J, Bumbarger, Perkins DJ, Williams D, Takeno M, Reid D, Castelli D, Sullivan D, Keenan T, Reid C, Da Costa NDa (2016) Linking functional and anatomical circuit connectivity using fast parallelized TEM imaging. Abstract Number: 186.04. Society for Neuroscience Annual Meeting (SFN).

[B3] Briggman KL, Bock DD (2012) Volume electron microscopy for neuronal circuit reconstruction. Curr Opin Neurobiol 22:154–161. 10.1016/j.conb.2011.10.022 22119321

[B4] Burns, R, Lillaney, K, Berger, DR, Grosenick, L, Deisseroth, K, Reid, RC, Roncal, WG, Manavalan, P, Bock, DD, Kasthuri, N, et al., (2013). The Open Connectome Project data cluster: scalable analysis and vision for high-throughput neuroscience. In Proc. of the 25th International Conference on Scientific and Statistical Database Management, 27 ACM. 10.1145/2484838.2484870 PMC3881956 24401992

[B5] Bushong EA, Johnson DD, Kim K-Y, Terada M, Hatori M, Peltier ST, Panda S, Merkle A, Ellisman MH (2015) X-ray microscopy as an approach to increasing accuracy and efficiency of serial block-face imaging for correlated light and electron microscopy of biological specimens. Microsc Microanal 21:231–238. 10.1017/S1431927614013579 25392009 PMC4415271

[B6] Chen F, Tillberg PW, Boyden ES (2015) Expansion microscopy. Science 347:543–548. 10.1126/science.1260088 25592419 PMC4312537

[B7] Chung K, Deisseroth K (2013) Clarity for mapping the nervous system. Nat Methods 10:508–513. 10.1038/nmeth.2481 23722210

[B8] Davis G, Mallat S, Avellaneda M (1997) Adaptive greedy approximations. Constr Approx 13:57–98. 10.1007/BF02678430

[B9] De Andrade V, Deriy A, Wojcik MJ, Gürsoy D, Shu D, Fezzaa K, De Carlo F (2016) Nanoscale 3d imaging at the advanced photon source. SPIE Newsroom May 12, 2016. doi:10.1117/2.1201604.006461.

[B10] De Carlo F, Gürsoy D, Marone F, Rivers M, Parkinson DY, Khan F, Schwarz N, Vine DJ, Vogt S, Gleber S-C, et al., (2014) Scientific data exchange: a schema for HDF5-based storage of raw and analyzed data. J Synchrotron Radiat 21:1224–1230. 10.1107/S160057751401604X 25343788

[B11] Deng J, Vine DJ, Chen S, Nashed YSG, Jin Q, Phillips NW, Peterka T, Ross R, Vogt S (2015) Simultaneous cryo X-ray ptychographic and fluorescence microscopy of green algae. Proc Natl Acad Sci USA 112:2314–2319. 10.1073/pnas.1413003112 25675478 PMC4345580

[B12] Donath T, Beckmann F, Schreyer A (2006) Automated determination of the center of rotation in tomography data. JOSA A 23:1048–1057. 16642181 10.1364/josaa.23.001048

[B13] Dowd, B. A., Campbell, G. H., Marr, R. B., Nagarkar, V. V., Tipnis, S. V., Axe, L., and Siddons, D. P. (1999). Developments in synchrotron X-ray computed microtomography at the national synchrotron light source . In SPIE ’ s International Symposium on Optical Science, Engineering, and Instrumentation, 224–236.

[B14] Eberle A, Mikula S, Schalek R, Lichtman J, TATE MK, Zeidler D (2015) High-resolution, high-throughput imaging with a multibeam scanning electron microscope. J Microsc 259:114–120. 10.1111/jmi.12224 25627873 PMC4670696

[B15] Economo MN, Clack NG, Lavis LD, Gerfen CR, Svoboda K, Myers EW, Chandrashekar J (2016) A platform for brain-wide imaging and reconstruction of individual neurons. eLife 5:e10566. 10.7554/eLife.10566 26796534 PMC4739768

[B16] Fratini M, Bukreeva I, Campi G, Brun F, Tromba G, Modregger P, Bucci D, Battaglia G, Spanò R, Mastrogiacomo M, et al., (2015) Simultaneous submicrometric 3D imaging of the micro-vascular network and the neuronal system in a mouse spinal cord. Sci Rep 5:8514. 10.1038/srep10771 PMC4649670 25686728

[B17] Gong H, Zeng S, Yan C, Lv X, Yang Z, Xu T, Feng Z, Ding W, Qi X, Li A, et al., (2013) Continuously tracing brain-wide long-distance axonal projections in mice at a one-micron voxel resolution. Neuroimage 74:87–98. 10.1016/j.neuroimage.2013.02.005 23416252

[B18] Gray Roncal W, Kleissas DM, Vogelstein JT, Manavalan P, Lillaney K, Pekala M, Burns R, Vogelstein RJ, Priebe CE, Chevillet MA, et al., (2015) An automated images-to-graphs framework for high resolution connectomics. Front Neuroinform 9:20. 10.3389/fninf.2015.00020 PMC4534860 26321942

[B19] Gray Roncal, W., Pekala, M., Kaynig-Fittkau, V., Kleissas, D. M., Vogelstein, J. T., Pfister, H., Burns, R., Vogelstein, R. J., Chevillet, M. A., and Hager, G. D. (2015). VESICLE: Volumetric Evaluation of Synaptic Inferfaces using Computer vision at Large Scale. In 26th British Machine Vision Conference (BMVC), 1–9.

[B20] Gürsoy D, De Carlo F, Xiao X, Jacobsen C (2014) Tomopy: a framework for the analysis of synchrotron tomographic data. J Synchrotron Radiat 21:1188–1193. 10.1107/S1600577514013939 25178011 PMC4181643

[B21] Heinzer S, Krucker T, Stampanoni M, Abela R, Meyer EP, Schuler A, Schneider P, Müller R (2006) Hierarchical microimaging for multiscale analysis of large vascular networks. Neuroimage 32:626–636. 10.1016/j.neuroimage.2006.03.043 16697665

[B22] Helmstaedter M, Briggman KL, Denk W (2011) High-accuracy neurite reconstruction for high-throughput neuroanatomy. Nat Neurosci 14:1081–1088. 10.1038/nn.2868 21743472

[B23] Hieber SE, Bikis C, Khimchenko A, Schweighauser G, Hench J, Chicherova N, Schulz G, Müller B (2016) Tomographic brain imaging with nucleolar detail and automatic cell counting. Sci Rep 6:32156. 10.1038/srep32156 PMC5007499 27581254

[B24] Howells MR, Beetz T, Chapman HN, Cui C, Holton JM, Jacobsen C, Kirz J, Lima E, Marchesini S, Miao H, Sayre D, Shapiro DA, Spence JCH, Starodub D (2009) An assessment of the resolution limitation due to radiation-damage in X-ray diffraction microscopy. J Electr Spectrosc Rel Phenom 170:4–12. 10.1016/j.elspec.2008.10.008 PMC2867487 20463854

[B25] Jasmin L, Burkey AR, Card JP, Basbaum AI (1997) Transneuronal labeling of a nociceptive pathway, the spino-(trigemino-) parabrachio-amygdaloid, in the rat. J Neurosci 17:3751–3765. 9133395 10.1523/JNEUROSCI.17-10-03751.1997 PMC6573681

[B26] Kak AC and Slaney M (1988). Principles of Computerized Tomographic Imaging. IEEE Press.

[B27] Karreman MA, Ruthensteiner B, Mercier L, Schieber NL, Solecki G, Winkler F, Goetz JG, Schwab Y (2017) Find your way with x-ray: using microct to correlate in vivo imaging with 3d electron microscopy. Methods Cell Biol 140:277–301. 28528637 10.1016/bs.mcb.2017.03.006

[B28] Kasthuri N, Hayworth KJ, Berger DR, Schalek RL, Conchello JA, Knowles-Barley S, Lee D, Vázquez-Reina A, Kaynig V, Jones TR, Roberts M, Morgan JL, Tapia JC, Seung HS, Gray Roncal W, Vogelstein JT, Burns R, Sussman DL, Priebe CE, Pfister H, Lichtman JW (2015) Saturated reconstruction of a volume of neocortex. Cell 162:648–661. 10.1016/j.cell.2015.06.054 26232230

[B65] Kleissas DM, Hider Jr. R, Gion T, Pryor D, Manavalan P, Baden A, Lillaney K, Burns R, Matelsky J, D’Angelo D, Gray Roncal W, Wester B “The Block and Object Storage System,” 2016. [Online]. Available: https://docs.theboss.io/. [*Accessed: 1-Sep-2017*].

[B29] Kreutzberg GW (1984) 100 years of Nissl staining. Trends Neurosci 7:236–237. 10.1016/S0166-2236(84)80213-1

[B30] Lapi D, Colantuoni A (2015) Remodeling of cerebral microcirculation after ischemia-reperfusion. J Vasc Res 52:22–31. 10.1159/000381096 25896412

[B31] Li A, Gong H, Zhang B, Wang Q, Yan C, Wu J, Liu Q, Zeng S, Luo Q (2010) Micro-optical sectioning tomography to obtain a high-resolution atlas of the mouse brain. Science 330:1404–1408. 10.1126/science.1191776 21051596

[B32] Lichtman JW, Denk W (2011) The big and the small: challenges of imaging the brain’s circuits. Science 334:618–623. 10.1126/science.1209168 22053041

[B33] Loftsgaarden DO, Quesenberry CP, et al., (1965) A nonparametric estimate of a multivariate density function. Ann Math Stat 36:1049–1051.

[B34] Madisen L, Zwingman TA, Sunkin SM, Oh SW, Zariwala HA, Gu H, Ng LL, Palmiter RD, Hawrylycz MJ, Jones AR, et al., (2010) A robust and high-throughput Cre reporting and characterization system for the whole mouse brain. Nat Neurosci 13:133–140. 10.1038/nn.2467 20023653 PMC2840225

[B35] Mikula S, Denk W (2015) High-resolution whole-brain staining for electron microscopic circuit reconstruction. Nat Methods 12:541–546. 10.1038/nmeth.3361 25867849

[B36] Mitra PP (2014) The circuit architecture of whole brains at the mesoscopic scale. Neuron 83:1273–1283. 10.1016/j.neuron.2014.08.055 25233311 PMC4256953

[B37] Mizutani R, Suzuki Y (2012) X-ray microtomography in biology. Micron 43:104–115. 10.1016/j.micron.2011.10.002 22036251

[B38] Mizutani R, Takeuchi A, Uesugi K, Takekoshi S, Osamura R, Suzuki Y (2010a) Unveiling 3D biological structures by X-ray microtomography. Microsc Sci Technol Appl Educ 379–386.

[B39] Mizutani R, Takeuchi A, Uesugi K, Takekoshi S, Osamura RY, Suzuki Y (2010b) Microtomographic analysis of neuronal circuits of human brain. Cereb Cortex 20:1739–1748. 10.1093/cercor/bhp237 19915092

[B40] Münch B, Trtik P, Marone F, Stampanoni M (2009) Stripe and ring artifact removal with combined wavelet-fourier filtering. Opt Express 17:8567–8591. 10.1364/OE.17.008567 19434191

[B41] Ng J, Browning A, Lechner L, Terada M, Howard G, Jefferis GS (2016) Genetically targeted 3D visualisation of Drosophila neurons under electron microscopy and x-ray microscopy using miniSOG. Sci Rep 6:38863. 10.1038/srep38863 PMC5153665 27958322

[B42] Oh SW, Harris JA, Ng L, Winslow B, Cain N, Mihalas S, Wang Q, Lau C, Kuan L, Henry AM, et al., (2014) A mesoscale connectome of the mouse brain. Nature 508:207–214. 10.1038/nature13186 24695228 PMC5102064

[B43] Paganin D, Mayo S, Gureyev TE, Miller PR, Wilkins SW (2002) Simultaneous phase and amplitude extraction from a single defocused image of a homogeneous object. J Microsc 206:33–40. 10.1046/j.1365-2818.2002.01010.x 12000561

[B44] Peng H, Hawrylycz M, Roskams J, Hill S, Spruston N, Meijering E, Ascoli GA (2015) Bigneuron: large-scale 3D neuron reconstruction from optical microscopy images. Neuron 87:252–256. 10.1016/j.neuron.2015.06.036 26182412 PMC4725298

[B45] Póczos, B. and Schneider, J. G. (2011). On the estimation of alpha-divergences . In Int. Conf. on Artificial Intelligence and Statistics, 609–617.

[B46] Qi X, Xing F, Foran DJ, Yang L (2012) Robust segmentation of overlapping cells in histopathology specimens using parallel seed detection and repulsive level set. IEEE Trans Biomed Eng 59:754–765. 22167559 10.1109/TBME.2011.2179298 PMC3655778

[B47] Rex DE, Ma JQ, Toga AW (2003) The loni pipeline processing environment. Neuroimage 19:1033–1048. 12880830 10.1016/s1053-8119(03)00185-x

[B48] Richardson DS, Lichtman JW (2015) Clarifying tissue clearing. Cell 162:246–257. 10.1016/j.cell.2015.06.067 26186186 PMC4537058

[B49] Rose A (1946) A unified approach to the performance of photographic film, television pickup tubes, and the human eye. J Soc Motion Picture Eng 47:273–294. 10.5594/J12772

[B50] Schindelin J, Arganda-Carreras I, Frise E, Kaynig V, Longair M, Pietzsch T, Preibisch S, Rueden C, Saalfeld S, Schmid B, et al., (2012) Fiji: an open-source platform for biological-image analysis. Nat Methods 9:676–682. 10.1038/nmeth.2019 22743772 PMC3855844

[B51] Sommer C, Strähle C, Köthe U, Hamprecht FA (2011) ilastik: Interactive Learning and Segmentation Toolkit Eighth IEEE International Symposium on Biomedical Imaging (ISBI). Proceedings, 230–233.

[B52] Tapia JC, Kasthuri N, Hayworth KJ, Schalek R, Lichtman JW, Smith SJ, Buchanan J (2012) High-contrast en bloc staining of neuronal tissue for field emission scanning electron microscopy. Nat Protoc 7:193–206. 10.1038/nprot.2011.439 22240582 PMC3701260

[B53] Timbie C, Barbas H (2015) Pathways for emotions: specializations in the amygdalar, mediodorsal thalamic, and posterior orbitofrontal network. J Neurosci 35:11976–11987. 10.1523/JNEUROSCI.2157-15.2015 26311778 PMC4549406

[B54] Tsai PS, Kaufhold JP, Blinder P, Friedman B, Drew PJ, Karten HJ, Lyden PD, Kleinfeld D (2009) Correlations of neuronal and microvascular densities in murine cortex revealed by direct counting and colocalization of nuclei and vessels. J Neurosci 29:14553–14570. 10.1523/JNEUROSCI.3287-09.2009 19923289 PMC4972024

[B55] Turaga SC, Murray JF, Jain V, Roth F, Helmstaedter M, Briggman K, Denk W, Seung HS (2010) Convolutional networks can learn to generate affinity graphs for image segmentation. Neural Comput 22:511–538. 10.1162/neco.2009.10-08-881 19922289

[B56] Weitkamp T, Haas D, Wegrzynek D, Rack A (2011) Ankaphase: software for single-distance phase retrieval from inline x-ray phase-contrast radiographs. J Synchrotron Radiat 18:617–629. 10.1107/S0909049511002895 21685680

[B57] Werner C, Engelhard K (2007) Pathophysiology of traumatic brain injury. Br J Anaesth 99:4–9. 10.1093/bja/aem131 17573392

[B58] Williams S, Zhang X, Jacobsen C, Kirz J, Lindaas S, van’t Hof J, Lamm SS (1993) Measurements of wet metaphase chromosomes in the scanning transmission X-ray microscope. J Microsc 170:155–165. 10.1111/j.1365-2818.1993.tb03335.x

[B59] Wu J, He Y, Yang Z, Guo C, Luo Q, Zhou W, Chen S, Li A, Xiong B, Jiang T, et al., (2014) 3D brainCV: simultaneous visualization and analysis of cells and capillaries in a whole mouse brain with one-micron voxel resolution. Neuroimage 87:199–208. 24185025 10.1016/j.neuroimage.2013.10.036

[B60] Yanagihara M, Niimi K, Ono K (1987) Thalamic projections to the hippocampal and entorhinal areas in the cat. J Comp Neur 266:122–141. 10.1002/cne.902660110 2448349

[B61] Yushkevich PA, Piven J, Cody Hazlett H, Gimpel Smith R, Ho S, Gee JC, Gerig G (2006) User-guided 3D active contour segmentation of anatomical structures: significantly improved efficiency and reliability. Neuroimage 31:1116–1128. 10.1016/j.neuroimage.2006.01.015 16545965

[B62] Zhang M-Q, Zhou L, Deng Q-F, Xie Y-Y, Xiao T-Q, Cao Y-Z, Zhang J-W, Chen X-M, Yin X-Z, Xiao B (2015) Ultra-high-resolution 3D digitalized imaging of the cerebral angioarchitecture in rats using synchrotron radiation. Sci Rep 5:14982. 10.1038/srep14982 PMC4595735 26443231

[B63] Zhang X, Jacobsen C, Lindaas S, Williams S (1995) Exposure strategies for polymethyl methacrylate from *in situ* X-ray absorption near edge structure spectroscopy. J Vacuum Sci Technol B 13:1477–1483. 10.1116/1.588175

[B64] Zilles K, Amunts K (2010) Centenary of Brodmann’s map: conception and fate. Nat Rev Neurosci 11:139–145. 10.1038/nrn2776 20046193

